# Emerging therapies to overcome antiandrogen resistance and beyond in lethal prostate cancer

**DOI:** 10.1016/j.jncc.2025.04.004

**Published:** 2025-05-24

**Authors:** Furong Huang, Kexin Li, Jeffrey W. Shevach, Qianben Wang

**Affiliations:** a Department of Pathology and Duke Cancer Institute Center for Prostate and Urologic Cancer, Duke University School of Medicine, Durham, United States; b Department of Medicine and Duke Cancer Institute Center for Prostate and Urologic Cancer, Duke University School of Medicine, Durham, United States

**Keywords:** Prostate cancer, Androgen receptor signaling, Therapy resistance, Precision medicine, Emerging therapies

## Abstract

Prostate cancer remains the second most common malignancy among men worldwide, with treatment paradigms evolving dramatically over the last two decades. Despite the longstanding efficacy of androgen deprivation therapy (ADT) and its combination with next-generation androgen receptor (AR) signaling inhibitors or chemotherapy in metastatic hormone-sensitive settings, most tumors ultimately develop resistance and progress to lethal castration-resistant prostate cancer (CRPC). This resistance often stems from a range of molecular alterations, including AR mutations, amplifications, splice variants, and tumor suppressor gene lesions (e.g., *TP53, RB1*). Recent advances in genomic and translational research underscore the importance of biomarker-guided patient stratification to optimize therapeutic choices. Novel strategies to circumvent resistance include non–ligand-binding-domain AR inhibitors, potent AR degraders (e.g., proteolysis-targeting chimeras [PROTACs]), bipolar androgen therapy, and combination regimens incorporating PARP inhibitors or immunotherapies for selected subsets of patients. Additionally, gene-editing approaches targeting “undruggable” genetic lesions offer promise in preclinical models. Moving forward, clinical development of these emerging agents and personalized treatment approaches, supported by robust genomic profiling, is poised to enhance tumor control, extend survival, and improve quality of life for patients with advanced prostate cancer.

## 1 Introduction

Prostate cancer (PCa) is the second most common cancer among men globally and a leading cause of cancer-related mortality worldwide.^1^ Its progression is fundamentally driven by androgen receptor (AR) signaling, which has led to the widespread use of androgen deprivation therapy (ADT) as the first-line approach for metastatic hormone-sensitive prostate cancer (mHSPC).^2^ Although ADT initially induces clinical regression, most tumors eventually evolve resistance and progress to lethal castration-resistant prostate cancer (CRPC).^2^ Over the past two decades, the treatment landscape of CRPC has expanded dramatically. The persistent dependence of CRPC on AR signaling paved the way for next-generation AR signaling inhibitors (e.g., enzalutamide and abiraterone), which have prolonged survival in many patients. While initially introduced and developed in the mCRPC setting, these agents—in combination with ADT with or without docetaxel chemotherapy—in the front-line mHSPC setting are now standard of care due to dramatically improved overall survival.^3^, ^4^, ^5^, ^6^, ^7^, ^8^, ^9^ However, recurrent genetic and epigenetic alterations that drive therapy resistance—such as AR mutations, amplifications, and splice variants, or tumor suppressor gene alterations (i.e., *TP53* and *RB1*)—have emerged as significant obstacles, limiting the long-term efficacy of these AR-targeting agents. Recent genomic and translational research has underscored the vital role of biomarker-guided patient stratification for optimizing therapeutic choices and improve outcomes.^10^^,^^11^ In this evolving context, new therapeutic options—such as PARP inhibitors (for tumors with homologous recombination [HR] defects), AKT pathway modulators (for PTEN-null disease), and immunotherapies (for patients with high microsatellite instability [MSI])—are offering promising results. Novel AR degraders (e.g., PROTACs) and inhibitors targeting AR non-ligand binding domains (LBD) (e.g., N-terminal domain [NTD], DNA-binding domain [DBD]) further highlight how mechanistic discoveries can translate into innovative drug design. This review integrates current standard-of-care practices with emerging research on resistance mechanisms and novel treatment strategies. Ultimately, leveraging comprehensive molecular profiling and multidisciplinary treatment paradigms may enable clinicians to personalize therapy, enhance tumor control, and prolong survival for patients with lethal prostate cancer.

## 2 Targeting androgen-AR signaling axis

**Fig. 1:**
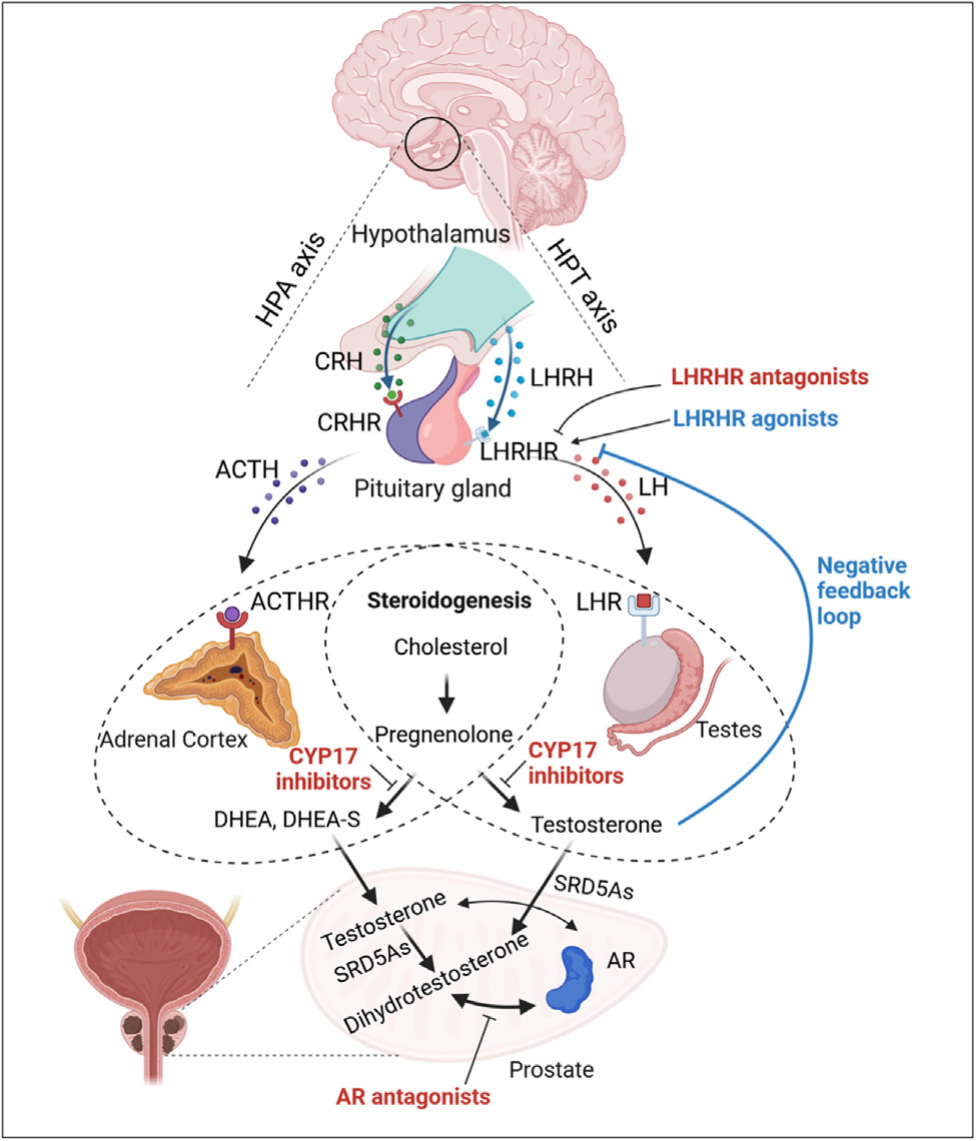
Mechanisms of action underlying androgen deprivation therapies and AR-targeted therapies. The biological synthesis of androgens (e.g., testosterone and the more potent DHT is regulated by both the HPT axis and the HPA axis. The hypothalamus secretes LHRH, which stimulates the pituitary gland to produce LH. LH then binds to its receptor (LHR) on Leydig cells in the testes, promoting testosterone production via the steroidogenesis pathway. Meanwhile, CRH secreted by the hypothalamus triggers production of ACTH from pituitary gland, which in turn stimulates the adrenal cortex to synthesize androgen precursors (e.g., DHEA and DHEA-S), ultimately converting them into testosterone. Testosterone can be further converted into DHT by SRD5As within the prostate or peripheral tissues. Although LHRH receptor agonists initially cause a transient increase in testosterone (‘flare’), prolonged administration initiates a negative feedback loop to inhibit LH release and reduce testosterone production. Similar effects may be achieved using LHRH receptor antagonists to directly block LH release. Inhibitors of CYP17, such as abiraterone, can effectively suppress adrenal, testicular, and intraprostatic androgen synthesis. Finally, AR antagonists hinder androgen signaling by preventing testosterone and DHT from binding the AR, thereby reducing AR transactivation. AR, androgen receptor; ACTH, adrenocorticotropic hormone; CRH, corticotropin-releasing hormone; CYP17, 17-hydroxy/17,20-lyase; DHEA, Dehydroepiandrosterone; DHT, dihydrotestosterone; HPA, hypothalamic–pituitary–adrenal cortex; HPT, hypothalamic–pituitary–testicular; LH, luteinizing hormone; LHRH, luteinizing hormone-releasing hormone; SRD5As, 5α-reductase enzymes. Created with Biorender.com.

Androgens, including testosterone and its more potent derivative dihydrotestosterone (DHT), are essential for organogenesis and growth of the prostate.^12^ Apart from its physiological functions during prostate development, the links between androgen and prostate cancer were first recognized over 80 years ago by Huggins and Hodges, who observed that removing the primary source of androgens through surgical castration (i.e., removal of the testes or adrenal glands) conferred a therapeutic benefit in men with prostate cancer.^13^ This seminal discovery greatly advanced our understanding of androgens’ roles in pathogenesis of prostate cancer, thereby establishing ADT as the cornerstone for prostate cancer treatment ever since.^2^ Subsequently, clarifying the role of the hypothalamic–pituitary–testicular (HPT) axis in androgen synthesis led to the implementation of one of the first endocrine therapy for prostate cancer: the use of high doses of estrogens. High-dose estrogens reduce the secretion of luteinizing hormone-releasing hormone (LHRH) (also known as gonadotropin releasing hormone [GnRH]) from the hypothalamus and luteinizing hormone (LH) from the pituitary gland, creating a negative feedback loop on the HPT axis that decreases androgen production.^14^^,^^15^ Given the adverse effects associated with estrogen therapy, the development of LHRH antagonists (e.g., degarelix, relugolix) or LHRH agonists (e.g., goserelin, leuprolide, buserelin), which target LHRH receptor in the pituitary gland, expanded the repertoire of ADT options to chemically modulate HPT axis, induce pharmacological castration, and ultimately promote PCa regression^16^, ^17^, ^18^(Fig. 1 and Table 1).

**Table 1:** Selected prostate cancer drugs approved by the U.S. Food and Drug Administration (FDA) on the market.

Drug category	Drug name	Condition	Target
Chemotherapy	Docetaxel	mHSPC, mCRPC	Microtubules
	Cabazitaxel	mCRPC	Microtubules
Radioligand therapy	Lutetium Lu 177 vipivotide tetraxetan	PSMA positive mCRPC	PSMA
	Radium-223 dichloride	mCRPC with symptomatic bone metastasis	NA
Androgen synthesis inhibitors	Abiraterone acetate	Very high-risk localized PC, mHSPC, mCRPC	CYP17A1
	Degarelix	Localized PC, mHSPC, mCRPC	GnRH analogs
	Relugolix		
	Leuprolide acetate		
	Goserelin acetate		
Second generation AR antagonists	Enzalutamide	mCRPC, nmCRPC, mHSPC, biochemical recurrence	AR
	Apalutamide	nmCRPC, mHSPC	
	Darolutamide	nmCRPC	
		mHSPC	
Novel targeted therapy	Olaparib	mCRPC with HRR gene mutations	PARP enzymes
	Rucaparib	mCRPC with mutations in the *BRCA1* or *BRCA2* gene	
	Talazoparib and enzautamide^19^	mCRPC with HRR gene mutations	
	Olaparib and abiraterone^20^	mCRPC with mutations in the *BRCA1* or *BRCA2* gene	
	Niraparib and abiraterone^21^	mCRPC with mutations in the *BRCA1* or *BRCA2* gene	
Cellular immunotherapy	Sipuleucel-T	mCRPC	Prostate acid phosphatase

Abbreviations: AR, androgen receptor; GnRH, gonadotropin-releasing hormone; HRR, homologous recombination repair; mCRPC, metastatic castration-resistant prostate cancer; mHSPC, metastatic hormone-sensitive prostate cancer; NA, not available; nmCRPC, non-metastatic castration resistant prostate cancer; PARP, poly(ADP-ribose) polymerase; PC, prostate cancer; PSMA, prostate specific membrane antigen.

The adrenal glands also play a significant role in androgen synthesis via the hypothalamic–pituitary–adrenal (HPA) axis, whereby the hypothalamic release of corticotropin–releasing hormone (CRH) stimulates the secretion of adrenocorticotropic hormone (ACTH) from the pituitary gland. ACTH, in turn, drives the production of androgen precursors, including dehydroepiandrosterone (DHEA) and DHEA sulfate (DHEA-S), which circulate at high levels in the bloodstream^22^^,^^23^(Fig. 1). Interestingly, PCa cells can take up these precursors from the bloodstream and convert them into active androgens^24^. 17α-hydroxyase/17,20-lyase (CYP17A1) is a critical enzyme in the androgen synthesis pathway, facilitating both adrenal production and intratumoral conversions of androgen precursors^25^^,^^26^. Capitalizing on this pathway, abiraterone was developed in the 1990s as a selective and potent CYP17 inhibitor. By inhibiting CYP17A1, abiraterone reduces serum androgen levels—particularly within PCa tissues—and has remained a standard therapy for advanced PCa^27^^,^^28^(Fig. 1 and Table 1).

The quest for complete androgen signaling blockade was significantly advanced by the discovery and characterization of AR, followed by the development of first-generation AR inhibitors, including flutamide, bicalutamide, and nilutamide(Fig. 1).^2^^,^^29^ Notably, monotherapy with these AR-targeted agents or their use in combination with ADT demonstrated anti-tumor activity and improved overall survival in treatment-​naive PCa patients.^30^^,^^31^ However, despite the initial clinical success of ADT and first-generation of anti-AR agents, the duration of response varied among patients, and most eventually developed resistance and progressed to CRPC, a lethal stage of the disease.^32^

First-generation AR inhibitors act by competing with endogenous androgen for binding to the AR’s LBD, thereby inhibiting AR-mediated transcriptional activity. However, these agents were reported to have lower binding affinity than endogenous androgens.^2^ Over the past few decades, advances in AR signaling research have led to the development of second-generation AR inhibitors, including enzalutamide,^5^^,^^6^^,^^33^ apalutamide^7^^,^^34^, and darolutamide^3^^,^^35^ (Table 1). These compounds display improved potency in suppressing multiple steps of AR signaling, including antagonizing androgen binding, preventing AR nuclear translocation, blocking AR-DNA binding, and limiting the recruitment of transcriptional co-activators. Although second-generation AR inhibitors have shown overall survival benefit in multiple clinical trials,^3^, ^4^, ^5^, ^6^, ^7^, ^8^, ^9^^,^^36^^,^^37^ acquired resistance to these more potent agents remain common. A comprehensive understanding of the underlying resistance mechanism is crucial for guiding the development of novel therapies to overcome AR inhibitor resistance and improve outcomes for patients with CRPC.

## 3 Molecular mechanisms of resistance to AR signaling inhibition and overcoming strategies

Despite the advances made with next-generation AR signaling inhibition, there are multiple common mechanisms of castration resistance, many of which involve persistent and/or aberrant AR signaling. These include AR genomic alterations and amplifications, alternative AR splicing events, aberrant AR transcriptional regulation, post-translational modification, glucocorticoid receptor-dependent AR bypass signaling, and circulating levels of non-testosterone androgens. Additionally, tumor suppressor gene loss and other genomic alterations can lead to the emergence of alternate CRPC molecular phenotypes through lineage plasticity or clonal selection, such as neuroendocrine CRPC, which do not typically respond to AR inhibition.

### 3.1 Diverse AR alterations and their contributions to therapy resistance

Resistance to molecularly targeted therapies such as ADT involves treatment-induced (epi)genomic alterations of target molecules, including genetic amplification and mutations,^38^^,^^39^ rewiring of functional/biological activities or deregulated stability of target proteins. AR amplifications were reported as early as 1995 in 30% of men who developed castration resistance following ADT^40^, which was associated with ADT failure in CRPC patients.^41^ AR amplifications partially account for an upregulation in AR expression.^42^^,^^43^ Antiandrogen treatment induces elevated *AR* mRNA and protein levels to confer therapy resistance by increasing the sensitivity to AR ligands and converting AR antagonists to exhibit agonistic activity through altering the recruitment and assembly of AR coregulators.^44^^,^^45^ Advances in PCa genomics extensively validated the higher frequency of AR amplifications in metastatic PCa and CRPC (about 50% of patients) compared with primary localized PCa (less than 8% of patients) across different cohorts.^46^, ^47^, ^48^, ^49^, ^50^, ^51^, ^52^, ^53^ In mCRPC, cell-free DNA (cfDNA) shows higher rates of AR amplification in patients treated with enzalutamide or abiraterone, which is indicative of treatment resistance and an increased risk of early disease progression.^54^, ^55^, ^56^, ^57^, ^58^, ^59^, ^60^ Additionally, mechanisms underlying increased AR expression include genomic amplification of a non-coding developmental enhancer located upstream of the *AR* gene locus (∼650 kb),^61^ which was clinically observed in 70%-87% of mCRPC cases.^62^ A recent study that performed AR-targeted DNA sequencing using CRPC and patient-derived xenografts (PDX) tissues showed AR was amplified on extrachromosomal DNA (ecDNA), representing another mechanism that explains the increased AR gene copies and expression under AR-targeted therapies.^63^

More than 1,000 AR point mutations have been identified, whereas only a few of them are reported to be associated with therapy resistance and PCa progression.^57^ Of note, the LBD is the primary target of both first- and second-generation AR antagonists and is recognized as a mutational hot spot in advanced PCa.^64^ The LBD harbors several recurrent and clinically significant point mutations, such as L702H, W742C, H875Y, F877L, and T878A, which collectively occur in 10%-30% of CRPC patients according to genomic sequencing studies, while being less frequently observed in early-stage disease.^65^^,^^66^ AR T878A was the first AR mutation site characterized in the human PCa cell line LNCaP,^67^ which, together with L702H and H875Y, were the most prevalent mutations based on a meta-analysis of 1,614 CRPC patients^68^ and two independent studies.^50^^,^^69^ These mutations have been observed in mCRPC patients before and after enzalutamide treatment,^55^ and are associated with AR promiscuity, enabling similar conformational changes and aberrant AR activation by non-canonical steroid ligands such as oestradiol (E2), adrenal androgens (e.g., DHEA), glucocorticoids, and progesterone.^70^, ^71^, ^72^, ^73^ Interestingly, AR W742C and F877L cause an antagonist-to-agonist switch, presumably by repositioning the carboxyl-terminal α-helix (helix 12) within the LBD,^74^, ^75^, ^76^ which enables AR activation rather than repression upon bicalutamide or enzalutamide treatment.^74^^,^^77^ While AR mutations are less frequently detected than AR amplification, their association with resistance to anti-androgen therapy has been well-documented.^55^^,^^57^ This resistance is most likely driven by mutation-induced structural alterations in AR that result in altered ligand selectivity and utilization.

To evade LBD-targeted anti-AR therapies, *AR* mRNA can also undergo aberrant alternative splicing, which generates about 20 truncated AR splice variants (AR-Vs) that lack the AR LBD and remain constitutively active in the absence of androgen ligands.^78^ AR-Vs are commonly detected in various preclinical PCa models^78^ and in patients with CRPC,^79^ and they have been recognized as another major mechanism of resistance to AR-targeted therapies, such as abiraterone and enzalutamide.^79^, ^80^, ^81^ Among AR-Vs, AR splice variant 7(AR-V7) has been extensively studied because AR-V7 is the most commonly expressed AR-V,^82^ and more importantly, detection of *AR-V7* mRNA in circulating tumor cells (CTC) increases substantially during progression to CRPC and closely correlates with resistance to either abiraterone or enzalutamide,^79^^,^^83^ indicating that AR-V7 expression may serve as a biomarker for anti-AR therapy response. The biogenesis of AR-V7 is partly induced by the redistribution of splicing factors as a consequence of ADT pressure^81^ or treatment with more potent AR inhibitors.^84^ Recent studies have also identified members of the 2-oxoglutarate-dependent JmjC domain–containing oxygenase family (e.g., JMJD6)^85^ as critical regulators of AR-V7 expression. AR-V7 has been reported to be co-expressed endogenously with full-length AR (AR-FL).^86^ It can function through accessing androgen responsive elements and driving AR signaling independent of AR-FL,^87^^,^^88^ activating AR-FL transcriptional activities,^86^ or co-binding with AR-FL to repress a subset of growth-suppressive genes.^89^ In addition, our study found that AR-V7 chromatin binding was heterogeneous among CRPC but universally governed by pioneer factor HoxB13.^90^ Moreover, AR-V7-mediated unique gene regulation required the cofactors ZFX and BRD4.^91^ Despite these efforts, some patients with AR-V7-null expression at mRNA or protein levels remained non-responsive to either enzalutamide or abiraterone.^92^ Furthermore, preclinical investigations have showed that AR-V7 overexpression alone did not confer resistance to castration or enzalutamide,^93^ suggesting that AR-V7 is necessary but may not be sufficient to drive castrate resistance in advanced prostate cancer. This highlights the need to consider additional AR structural alterations, such as AR amplification or mutations.^94^

Notably, AR genomic alterations, such as amplification and point mutations, are not mutually exclusive and can co-occur in CRPC patients. Whole-genome sequencing of 49 cfDNA samples collected from 21 mCRPC patients treated with AR signaling inhibitors revealed that nearly half of patients harbored simultaneous amplifications in *AR* gene locus and a previously characterized AR enhancer,^61^ and some of them carried missense mutations or truncating structural variants in AR LBD.^95^ Other studies also reported that co-occurrence of *AR* mutations and amplification has been observed in up to 33.3% of CRPC patients using cfDNA-based lipid biopsy.^96^, ^97^, ^98^ The co-occurrence of AR common alterations also exists in PCa derived cell lines such as VCaP, which harbors both AR amplification^99^ and splicing variants.^93^ In addition, a recent study reported that 15% of tumors from a cohort of PDXs and 23 CRPC metastases exhibited very high *AR* copy number along with multiply-rearranged *AR* gene structures.^63^ While AR-V7 is not a genomic alteration but an alternatively spliced transcript, recent studies suggest that *AR* gene amplification may enhance AR-V7 expression. In a cohort of 95 CRPC biopsies, AR-V7 protein expression was found significantly correlated with AR copy number (*r* = 0.23, *P* = 0.026).^100^ Moreover, levels of AR-V7 were significantly higher in tumors with an increase in *AR* gene copy number compared with tumors harboring a single *AR* gene copy.^101^ Collectively, these findings demonstrate the molecular complexity and heterogeneity of AR-related alterations and highlight how diverse mechanisms may converge to drive resistance to AR-targeted therapy in a subset of CRPC patients.

### 3.2 Transcriptional and post-translational control of AR

Interestingly, AR activity is highly dynamic and can become aberrantly manifested during prostate cancer progression or in response to AR inhibitors. Profiling of the genome-wide distributions of AR (i.e., AR cistromes) showed AR cistromes were extensively reprogrammed in primary PCa tissues compared to normal prostate,^102^ which were further redistributed in metastatic PCa.^103^^,^^104^ Alterations of AR cistromes were dependent on recruitment of AR-cofactors or chromatin regulators, such as pioneer factor FOXA1,^103^, ^104^, ^105^, ^106^ HoxB13^102^^,^^103^^,^^107^ or GATA2,^104^^,^^106^^,^^108^, ^109^, ^110^ histone acetylation reader BRD4^111^ or writer CBP/P300,^112^ and chromatin remodelers SWI/SNF complex^113^ and CHD1.^114^ Several transcriptional coactivators of AR, such as p160 steroid receptor coactivators^47^^,^^110^ and the Mediator complex (e.g., MED1),^115^, ^116^, ^117^, ^118^ were found to be aberrantly overexpressed to maximize AR signaling and drive castration resistance. Unexpectedly, our previous studies demonstrated that antagonist-liganded AR bound to a distinct DNA motif compared with agonist-liganded AR, which was facilitated by GATA2 and the Mediator complex to directly orchestrate the transcription of a group of oncogenes, thereby driving resistance to AR antagonists.^119^^,^^120^ Recent studies have nominated novel AR co-activators in governing AR-driven luminal lineage,^121^ mediating AR chromatin occupancy, therapy resistance and prostate tumorigenesis.^122^ These findings suggest targeting these AR cofactors and chromatin regulators may serve as an alternative strategy to counteract AR oncogenomic functions and overcome resistance to AR-targeted therapy. Notably, the ectopic expression of both FOXA1 and HoxB13 in an immortalized prostate epithelial cell line (LHSAR) reprogrammed AR cistromes into a prostate tumor-like state,^102^ and in metastatic PCa, AR binding sites were pre-occupied by FOXA1 and HoxB13,^103^ highlighting their oncogenic roles in governing AR transcriptional regulation during PCa tumorigenesis and metastasis. However, the functional analysis of FOXA1 and HoxB13 in CRPC has yielded conflicting findings. While the majority of studies have reported that these factors exhibit higher protein expression in CRPC metastatic samples^123^^,^^124^ and promote cell growth,^90^^,^^125^ invasion, and *in vivo* metastasis,^126^ a few studies have suggested their tumor-suppressive roles in prostate cancer.^107^^,^^127^ This controversy may arise from the context-dependent functions of these transcription factors and from differences in loss-of-function approaches used across studies—ranging from siRNA/shRNA (which may have significant off-target effects) to CRISPR/Cas13 (offering improved specificity). For instance, despite the role of HoxB13 in the transcriptional upregulation of AR^128^ and in directing genomic functions of both AR^102^^,^^103^ and AR-V7^90^ in CRPC, HoxB13 depletion may lead to an alternative pathway of resistance through upregulation of fatty acid synthase FASN and increased *de novo* lipogenesis.^107^ Further research is needed to validate these findings and evaluate the feasibility and efficacy of targeting FOXA1 and HoxB13 as a therapeutic strategy in CRPC.

The transcriptional activities of AR are also regulated by post-translational mechanisms through AR protein modifications, including phosphorylation, acetylation, methylation, ubiquitination, and SUMOylation, which impact protein stability, nuclear localization and transcriptional activity of AR.^129^ While transcriptional activities of AR can be modulated by various post-translational modifications (PTMs), ubiquitination-mediated AR degradation has received increasing research attention due to its direct link to AR protein stability.^129^ One of the best-characterized mechanisms involves SPOP, a substrate-recognition subunit of the cullin-3 (CUL3)–RING-box 1 (RBX1) E3 ubiquitin ligase (CRL) complex. SPOP functions as a tumor suppressor by targeting AR for ubiquitination and proteasomal degradation.^130^, ^131^, ^132^ However, SPOP is frequently mutated in primary PCa, impairing its ability to degrade AR, resulting in elevated AR protein levels.^132^^,^^133^ While SPOP alterations confer resistance to androgen deprivation monotherapy, recent retrospective studies have shown that the use of modern AR signaling inhibitors in the first-line mHSPC treatment setting (i.e. abiraterone, enzalutamide, etc.) is associated with greater progression-free and overall survival due to the addiction of these cancers to AR activity.^134^ In addition to SPOP, Siah2 has been reported as an AR-targeting E3 ligase that specifically recognizes the transcriptionally inactive, co-repressor NCOR1-bound form of AR for ubiquitin-dependent degradation, thereby promoting prostate cancer progression in castrated conditions.^135^ On the other hand, deubiquitinase USP11 was shown to stabilize AR protein, providing a mechanism of enhanced AR signaling in CRPC.^136^ Recent studies have expanded the spectrum of AR ubiquitination sites and their functional consequences. In particular, Arai et al.^137^ identified a previously unreported ubiquitination site at K911, located near the AR carboxy terminus, where mutation of K911 enhanced AR stability, chromatin binding, and transcriptional activity, suggesting this site plays a role in controlling AR turnover on chromatin. In addition, K313, another previously identified ubiquitination site, was found to also undergo methylation and acetylation, indicating that multiple types of PTMs can converge at specific residues to fine-tune AR transcriptional output. These findings highlight the complex interplay of PTMs in regulating AR degradation and activity, and their potential contributions to therapeutic resistance in prostate cancer.^137^

### 3.3 Persistent androgen production and bioavailability despite castration

Notably, in addition to multifaceted alterations in *AR* gene and/or proteins or regulatory activities, elevated AR ligand levels also contribute to restored or sustained AR signaling even during treatment with potent AR signaling inhibitors such as abiraterone and enzalutamide in CRPC^2^. While medical or chemical castration can reduce the serum testosterone levels by over 90%,^138^ residual androgens such as DHEA and androstenedione (AD), produced primarily by the adrenal glands via *de novo* steroidogenesis, continue to serve as major substrates for intraprostatic synthesis of testosterone and DHT.^2,^^139^ Despite the ability of abiraterone to block CYP17A1 and lower DHEA and AD, sulfated DHEA (S-DHEA) persists in circulation and remains a key precursor for DHT synthesis within prostate tissues.^140^ In this way, tumors can circumvent castration-level androgens through intracrine mechanisms.

Moreover, aberrant overexpression^141^ (e.g., CYP17A1, 5α-reductase type 1 [SRD5A1]) or gain-of-function mutations (e.g., 3β-hydroxysteroid dehydrogenases 1 [3β-HSD1]^142^) in critical steroidogenic enzymes further enhance intra-tumoral androgen production, sustaining high DHT levels that drive CRPC growth.^143^ Of particular example is the 3β-HSD1 1245A>C polymorphism, which leads to increased extragonadal synthesis of androgens,^142^ and has been associated with worse clinical outcomes in patients receiving AR signaling inhibitors in multiple studies.^143^^,^^144^ Additionally, in patients receiving abiraterone, the 1245A>C single nucleotide polymorphism is associated with an increase in the drug metabolite 3-keto-5α-abiraterone, which can stimulate the AR and diminish abiraterone’s efficacy.^143^ Persistent androgen synthesis and aberrant drug metabolisms through common genetic alterations effectively undermines the therapeutic benefit of abiraterone and enzalutamide by maintaining AR signaling, highlighting a key mechanism of treatment resistance.

Collectively, these pathways underscore the importance of inhibiting or bypassing residual androgen biosynthesis to improve clinical outcomes. Strategies targeting steroidogenic enzymes beyond CYP17A1—such as 3β-HSD inhibitors or next-generation 5α-reductase inhibitors—are under investigation to block DHT formation more comprehensively. Such approaches are especially relevant in tumors that evolve compensatory steroidogenic routes to sustain androgen availability. Ultimately, understanding these mechanisms of androgen bioavailability, pharmacokinetics and pharmacodynamics enables more precise targeting of steroid hormone synthesis in CRPC, optimizing existing therapies and guiding the development of novel agents that can overcome or delay treatment resistance.

### 3.4 GR-dependent AR bypass signaling and paradoxical glucocorticoid effects

Instead of complete AR independence as historically described,^145^^,^^146^ AR bypass signaling is now recognized as a glucocorticoid receptor (GR)-dependent mechanism by which enzalutamide-resistant prostate cancer cells reactivate AR transcriptional programs. This occurs because the GR cistrome and downstream gene networks overlap substantially with those of AR, allowing GR activation (e.g., by dexamethasone) to restore AR target gene expression and promote therapeutic resistance.^147^ Notably, blocking GR with antagonists or silencing GR genetically can reverse this resistance.^147^ Clinical studies further showed that GR expression is upregulated following treatment with enzalutamide or chemical castration plus abiraterone in prostate cancer.^148^^,^^149^ Mechanistically, AR represses GR expression through direct binding to the *NR3C1* (encoding GR) locus^139^; however, in the presence of enzalutamide, AR binding at distal regulatory elements of *NR3C1* may instead promote its expression.^120^ This GR-mediated AR bypass can seem paradoxical given that synthetic glucocorticoids (e.g., prednisone, dexamethasone) are routinely used in prostate cancer—both as monotherapy to reduce pituitary ACTH production (thus lowering androgen levels) and to counteract side effects of other treatments.^150^^,^^151^ For example, prednisone is often co-administered with docetaxel chemotherapy to reduce toxicities^150^^,^^152^ and is mandatory with abiraterone to prevent hypertension and hypokalemia arising from compensatory increases in mineralocorticoids.^150^^,^^152^ However, in tumors that exhibit high GR expression, these same glucocorticoids may promote therapy resistance via GR-driven reactivation of AR-like transcriptional programs.^139^ Beyond GR, other steroid receptors such as progesterone receptor (PR) share significant structural homology in their DNA-binding domains, enabling them to bind similar regulatory elements and contribute to AR bypass.^153^^,^^154^

Taken together, these observations underscore the complexity of glucocorticoid use in prostate cancer management. While glucocorticoids reduce adrenal androgen production and mitigate adverse effects associated with chemotherapy or abiraterone-induced mineralocorticoid excess, they can simultaneously drive an alternative GR-dependent pathway of AR signaling, especially in cancers with elevated GR. Novel therapeutic approaches—such as selective GR modulators or combination regimens that more selectively target the GR axis—are under investigation and may help preserve the advantages of glucocorticoids while minimizing resistance.

### 3.5 Lineage plasticity and emergence of neuroendocrine and other CRPC subtypes

Accumulating evidence suggests that persistent selective pressure following potent AR inhibitors (e.g., enzalutamide or abiraterone) induces the lineage transition of a subset of CRPC tumors (20%–25%) from AR-dependent to an AR-independent state. This transition is accompanied by hybrid or complete acquisition of neuronal or neuroendocrine (NE) features, such as distinct tumor cell histology and expression of neuroendocrine markers (e.g., synaptophysin and chromogranin),^11^^,^^155^ representing another important mechanism of AR-targeted therapies resistance via lineage switching. Molecularly, these AR-independent CRPC-NE tumors often exhibit loss of canonical AR activities or absence of AR expression; loss of tumor suppressor genes *TP53* (56%-67%)^156^^,^^157^ and *RB1*(70%-90%)^156^^,^^157^; *MYCN* amplification (∼40%)^158^; *ERG* rearrangements (∼50%)^159^; phenotypic switching from AR-expressing luminal cells to basal-like cells with NE marker expression; and upregulation of the pluripotent transcriptional factor (TF) *SOX2*^160^ and the epigenetic regulator *EZH2.*^11^^,^^161^ While histologically classified CRPC-adeno and CRPC-NE tumors share substantial overlaps in genomic alterations,^157^ the co-occurrence of *TP53* and *RB1* loss significantly increases in CRPC tumor with neuroendocrine differentiation compared to CRPC-adeno tumors (74% vs. 39%^160^ or 53% vs. 13%),^50^^,^^157^ which is dramatically associated with highly aggressive clinical features, resistance to AR pathway inhibitors, and shorter time to progression. Epigenetic reprogramming is a major driver of neuroendocrine differentiation. Typically, marked differences in genome-wide methylation are observed between CRPC-adeno and CRPC-NE tumors, and differentially methylated genes are enriched for neuronal and developmental pathways in CRPC-NE tumors.^157^ Additional lineage-associated factors have been found to drive emergence and progression of neuroendocrine prostate cancer (NEPC) or anti-androgen treatment induced trans-differentiation of luminal cells, including increased expression or activation of TFs such as N-Myc,^162^ Onecut2,^163^ ACSL1/2,^164^ or BRN2,^165^ as well as chromatin remodeler SWI/SNF complex.^166^ Furthermore, beyond the well-characterized CRPC-NE, recent epigenomic and transcriptomic profiling of CRPC organoids, PDXs, and cell lines has defined other lineages, including a Wnt-dependent subtype (CRPC-WNT) and a stem cell–like (SCL) subtype (CRPC-SCL).^105^ Moreover, an additional CRPC subtype derived from the treatment-induced lineage plasticity process is double-negative prostate cancer (DNPC), a subset of CRPC that lacks both the morphological features and IHC markers of NEPC, as well as AR expression.^167^ The molecular heterogeneity of CRPC underscores the importance of precise classification of CRPC patients, which could potentially guide the development of novel targeted therapies and inform treatment decisions.

## 4 Emerging therapies for PCa

### 4.1 Revisiting AR signaling for exploring potential targetable vulnerability

Treatment options for prostate cancer often rely on precise clinical staging or biomarkers-based molecular stratification, which collectively involve multidisciplinary efforts, such as biochemical testing of prostate-specific antigen, histopathological evaluation, and genomic testing using tumor or liquid biopsies.^168^ While clinically localized prostate cancer is commonly treated with surgery (e.g. radical prostatectomy) and/or radiation therapy (with or without ADT), advanced stages of diseases—encompassing mHSPC, non-metastatic CRPC, or mCRPC—is primarily managed by ADT and potent second-generation AR signaling inhibitors.^10^ Although resistance to AR signaling inhibition invariably occurs, these resistance mechanisms are often found in the AR signaling pathway. Thus, there are ongoing efforts to investigate alternative hormonal-based approaches and develop newer AR-targeted agents to take advantage of continued dependence on AR signaling in the majority of mCRPC.

### 4.2 Newer generation of AR-targeted agents

#### 4.2.1 Novel AR inhibitors

**Fig. 2:**
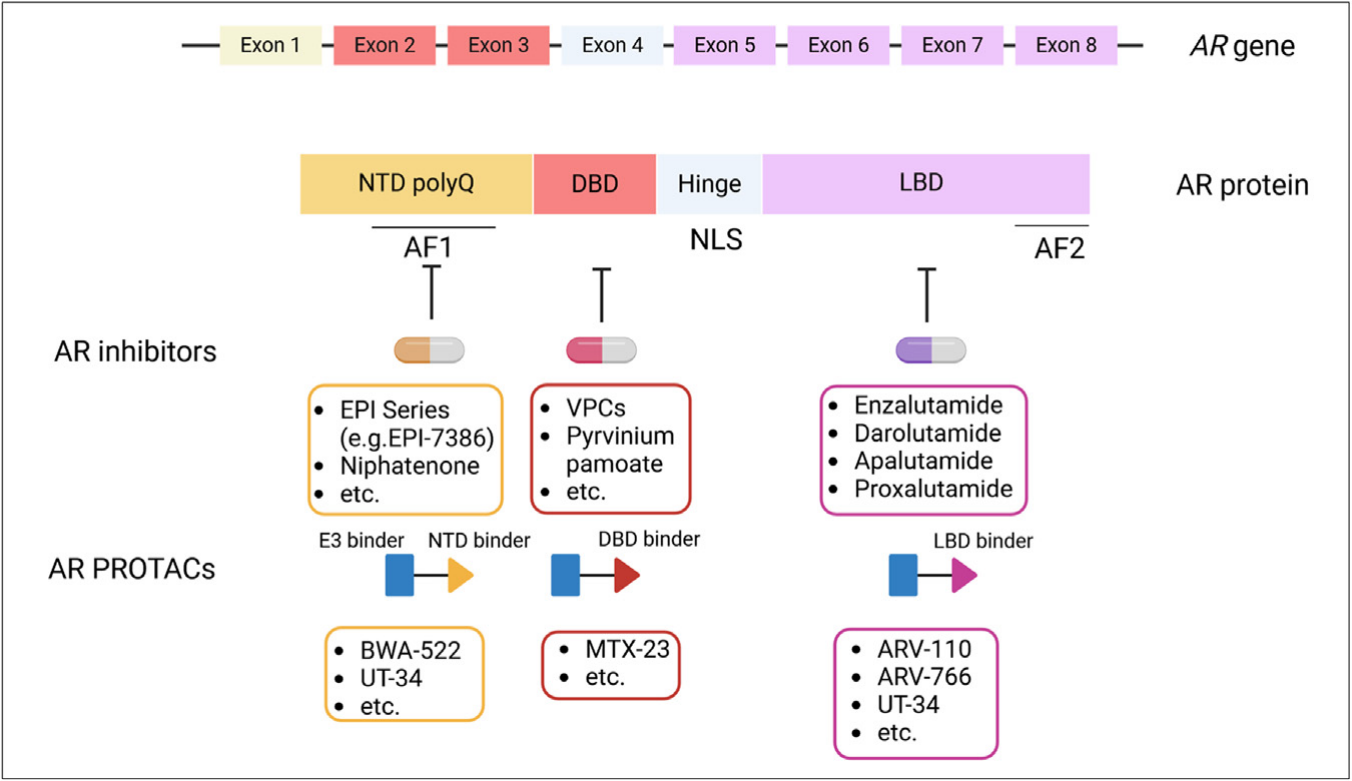
Development of novel AR inhibitors and AR PROTACs. In addition to conventional AR inhibitors directly against the AR LBD domain—which often acquires resistance-associated alterations—novel inhibitors targeting the AR NTD or DBD have been developed with distinct mechanisms of action and the potential to overcome resistance to current therapies. Emerging AR PROTACs, which function as heterobifunctional molecules, recruit the AR protein to an E3 ligase (e.g., via Von Hippel–Lindau [VHL] or cereblon [CRBN]-binding anchors) for ubiquitination and proteasomal degradation. The AR-binding moieties in these PROTACs are often derived from known AR antagonists or newly developed domain-specific inhibitors (UT-34 is considered a “pan-AR degrader”). Notably, many LBD-targeted AR PROTACs constitutes the largest category and are already under evaluation in early-phase of clinical trials for castration-resistant prostate cancer. AF, transcriptional activating function; AR, androgen receptor; DBD, DNA-binding domain; LBD, ligand binding domain; NLS, nuclear localiszation signal; NTD, N-terminal domain; PROTAC, proteolysis-targeting chimera. Created with Biorender.com.

To combat the resistance mechanisms underlying current FDA-approved AR-targeted therapies (Table 1), multiple new AR inhibitors with distinct mechanisms of action have been developed and evaluated. A notable example is Proxalutamide (GT-0918), a novel AR antagonist with higher binding affinity than enzalutamide, capable of antagonizing both wild-type AR and clinically observed missense mutations (i.e. F877L, W747C, and H875Y). GT-0918 has shown a well-tolerated safety profile and is currently being evaluated in a Phase II study in mCRPC patients who progressed after either abiraterone or enzalutamide(Table 2).^169^ Similarly, darolutamide—a recently approved AR antagonist—maintains antagonistic effects against multiple AR mutations.^170^ However, both darolutamide and GT-0918 fail to target constitutively active AR splice variants; furthermore, continuously evolving AR mutations may confer ultimate resistance to these drugs. Hence, agents targeting non-LBD domains represent alternative and promising strategies to overcome drug resistance regardless of LBD status (Fig. 2).

**Table 2:** Emerging therapies in clinical trials of prostate cancer.

Drug name	Drug category	Target	Patient eligibility	Clinical setting	Trials identifier	Results
Testosterone and Radium-223	BAT backbone	AR	mCRPC with bone metastases	Phase 2	NCT04704505	Ongoing
ARV-766	PROTAC degrader		mHSPC, mCRPC	Phase 1/2	NCT05067140^171^	Ongoing
Proxalutamide (GT0918)	AR-LBD antagonist		mCRPC	Phase 2/3	CTR20170177^172^	GT0918 showed a manageable safety profile and preliminary anti-tumor activity in patients with mCRPC
TRC-253			mCRPC	Phase 1/2A	NCT02987829^173^	TRC-253 daily at 280 mg was well-tolerated and selected as phase 2 dose
Ipatasertib or Apitolisib	AKT inhibitor	AKT	mCRPC pre-treated with Docetaxel	Phase 1b/2	NCT01485861^174^	In mCRPC, combined blockade with abiraterone and ipatasertib showed superior antitumor activity to abiraterone alone, especially in patients with PTEN-loss tumors.
Capivasertib			mHSPC and PTEN deficiency used with abiraterone	Phase 3	NCT04493853^175^	Awaiting results
Saruparib	PARP inhibitor	PARPs	mHSPC, used with enzalutamide, abiraterone or darolutamide	Phase 3	NCT06120491^176^	Ongoing
Fuzuloparib			mCRPC, used with abiraterone	Phase 3	NCT04691804	Ongoing
Tarlatamab	BiTE	DLL3	NEPC	Phase 1	NCT04702737^177^	Modest single-agent efficacy; slightly greater efficacy in DLL3^+^ tumors (by immunohistochemistry); manageable toxicity
Xaluritamig	BiTE	STEAP1	mCRPC	Phase 1	NCT04221542^178^	24% objective response rate at all dose levels; 41% objective response rate at higher dose levels
				Phase 3	NCT06691984	Ongoing
Enoblituzumab	B7-H3 monoclonal antibody	B7-H3	Localized prostate cancer, neoadjuvant prior to prostatectomy	Phase 2	NCT02923180^179^	Reasonably well tolerated and potential clinical activity^179^
Mevrometostat	EZH2 inhibitor	EZH2	mCRPC used with enzalutamide	Phase 3	NCT06629779^180^	Ongoing
			mCRPC used with enzalutamide post-abiraterone	Phase 3	NCT06551324^181^	Ongoing

Abbreviations: AR, androgen receptor; BAT, bipolar androgen therapy; BiTE, bispecific T-cell engager; LBD, ligand binding domain; mCRPC, metastatic castration-resistant prostate cancer; mHSPC, metastatic hormone-sensitive prostate cancer; PARP, poly(ADP-ribose) polymerase; PROTAC, proteolysis-targeting chimera.

The AR NTD is intrinsically disordered region encoded by exon 1, containing the activating function 1 (AF1) segment, which is essential for AR transactivation both in the presence and absence of ligand.^2^ It also mediates coactivator interactions, including the p160 steroid receptor coactivator (SRC) family (SRC-1, SRC-2, and SRC-3) and CBP/p300.^182^ An AF-1 antagonist, EPI-001, was first identified in 2010 and blocks transactivation of the NTD by inhibiting protein-protein interactions with AR and reducing AR binding to the androgen response elements, thereby suppressing CRPC growth.^183^ Several EPI analogues have since been developed that effectively overcome multiple drivers of aberrant AR activity, including overexpressed coactivators, AR gain-of-function mutations, and constitutively active AR splice variants.^182^^,^^184^ Moreover, EPI-mediated inhibition of AR is not affected by polymorphic polyglutamine lengths in the NTD.^182^ While the first-in-human EPI-506 ended due to high pill burden,^185^ EPI-7386 (masofaniten), a more potent EPI derivative with increased metabolic stability, is currently in a Phase I clinical trial^186^(NCT04421222). EPI treatment also overcomes enzalutamide resistance in cultured cells,^187^ and potential therapeutic synergy between EPI-7386 and enzalutamide is being investigated in mCRPC patients in a Phase I/II clinical trial^186^ (NCT05075577). Additional small molecules targeting the NTD include Niphatenone, Sintokamides, IMTPPE, QW07, VPC-220010, and EIQPN, identified through cell-based assay or structure-based screenings, along with ASR-600 and SC428 (inhibitors targeting AR-Vs), as well as bispecific antibodies.^188^ Importantly, the disordered NTD plays a predominant role in the phase separation of AR, which controls AR stability and transcriptional activities.^189^ A phenotypic screen leveraging phase-separation properties identified ET516, which targets AF-1 to disrupt AR condensates, blocks AR transactivation, and inhibits the growth of prostate cancer cells expressing resistant AR mutants.^190^

The AR DBD is encoded by exons 2 and 3 and contains two zinc finger motifs: (1) the P-box ‘recognition helix’, which defines DNA binding specificity, and (2) the D-box, facilitating receptor dimerization and stabilization of the DNA-receptor complex.^2^^,^^191^ Compounds targeting the DBD of AR can block AR binding to DNA and/or disrupt its homodimerization, thus inhibiting AR activation and cancer progression independent of LBD alterations.^186^ Pyrvinium pamoate (PP) was the first small-molecule AR DBD inhibitor; evidence shows that PP blocks AR signaling and prevents RNA polymerase II recruitment.^192^^,^^193^ Potential off-target effects of PP have been observed due to its crosstalk with other nuclear hormone receptors (e.g., estrogen or glucocorticoid receptors), which may paradoxically help counteract GR-dependent bypass signaling.^193^ Subsequently, VPC compounds demonstrated higher specificity for the AR DBD compared with PP.^186^^,^^194^ VPC-14449 binds the P-box region of the AR DBD and reduces AR chromatin binding, whereas VPC-17005 targets the D-box of DBD to block AR dimerization.^194^^,^^195^ Both agents suppress CRPC cell viability and growth while inhibiting AR transactivation.

#### 4.2.2 AR PROTACs

Targeted AR protein degradation is gaining recognition as a promising therapeutic modality in CRPC treatment.^196^ AR degraders include AR-targeting proteolysis-targeting chimeras (PROTACs), selective AR degraders (SARDs), and molecular glue degraders.^196^ Protein degraders offer potential advantages over traditional small-molecule inhibitors, including the abilities to degrade the non-enzymatic “undruggable” proteins, iteratively and durably eliminate targets, a non-occupancy-driven mechanism, and a potentially broader therapeutic window.^197^^,^^198^ A PROTAC is a heterobifunctional molecule that bridges an ubiquitin E3 ligase and a target protein to form a ternary complex, thereby inducing polyubiquitination and degradation of the substrate^197^^,^^198^ AR PROTACs have been developed to target the LBD, DBD or NTD (Fig. 2). While LBD alterations are major drivers of AR-targeted therapy resistance, LBD-based AR PROTACs remain widely pursued, as they rely on AR binders derived from known antagonists or selective AR modulators (SARMs).^196^ Typically, these binders are linked to an E3 ligase-recruiting anchor (e.g., a Von Hippel–Lindau [VHL] ligand^199^ or a cereblon [CRBN]-binding moiety).^200^ Compared with VHL-based molecules, many CRBN-based PROTACs offer improved pharmacokinetics and oral bioavailability.^196^ Notably, several AR LBD PROTACs have advanced into clinical trials and shown promising preliminary results. ARV-110 was the first-in-human PROTAC, leveraging an LBD antagonist as the warhead to recruit AR to the Cullin–RING ligase 4–cereblon (CRL4–CRBN) ligase complex.^197^ In a Phase I/II trial, ARV-110 demonstrated an acceptable safety profile and anti-tumor activity in mCRPC patients pretreated with enzalutamide or abiraterone, including those harboring AR T878A or H875Y mutations.^201^^,^^202^ Several other AR LBD PROTACs, such as ARV-766, CC-94676, HP-518, AC-0176, RO7656594, are likewise in Phase I trials for mCRPC (Table 2). Among these, ARV-766 exhibits an improved safety profile, higher stereochemical stability, and stronger degradation potency against prevalent AR mutants (e.g., L702H, H875Y, and T878A).^203^^,^^171^ Additional VHL-based or CRBN-based AR LBD PROTACs (e.g., ARCC4^199^, ARD-1676)^204^ display increased potency, broader coverage of AR mutants, and high oral bioavailability, highlighting the potential for further clinical evaluation.

Similar to the DBD or NTD-targeted novel AR inhibitors described above, AR DBD or NTD PROTACs can degrade not only wild-type AR but also AR harboring point mutations or LBD-truncated AR-Vs, offering a route to overcome resistance independent of the LBD (Fig. 2).^196^ Notably, DBD or NTD binders originate from currently available DBD or NTD targeting inhibitors.^196^ For instance, MTX-23—the first-in-class AR DBD PROTAC—uses VPC-14449 analogs as DBD binders.^205^ EPI-series molecules have also been leveraged to develop NTD binders, culminating in BWA-522, the first AR NTD PROTAC.^206^ Additional AR degraders include SARDs, which facilitate AR degradation by engaging MDM2 (e.g., galeterone and derivatives VNPP433–3b or ASC-J9)^196^ or by directly binding the LBD or NTD to induce AR conformational changes leading to degradation (e.g., UT series SARDs).^207^ UT-34, a pan AR degrader capable of inhibiting wild-type, LBD-mutant, and AR-Vs both *in vitro* and *in vivo,*^208^ has entered a Phase I/II clinical trial in mCRPC patients (NCT05917470).

### 4.3 Bipolar androgen therapy

Paradoxically, the growth proliferative effects of testosterone on AR-expressing prostate cancer cells were found not to act in a dose-dependent manner.^209^ Previous studies^210^^,^^211^ and a recent report^212^ have showed that LNCaP or VCaP cells exhibited a biphasic response to testosterone, where low-dose synthetic androgen (i.e., R1881) stimulated growth, while high-dose androgen at supraphysiological levels (≥ 1 nM R1881), dramatically inhibited cell proliferation *in vitro*, a non-linear dose response to androgens that was also recapitulated *in vivo.* The growth inhibition by supraphysiological T was dependent on AR expression in PCa cells,^213^ and extensive investigations suggest that this effect can be attributed to decreased Myc expression at both RNA and protein levels, accompanied by increased p21 and p27 associated with G1 cell cycle arrest.^212^^,^^214^ Interestingly, AR can interact with DNA replication licensing factors, and supraphysiological T prevents AR degradation, resulting in accumulated AR-licensing factors complex in origins of replications, thus blocking relicensing and inducing cell cycle arrest.^215^^,^^216^ Similarly, high-dose androgen modulates the RB-E2F1 pathway by inhibiting RB phosphorylation and repressing cell cycle genes.^217^ Additional mechanisms underlying growth suppression involve the induction of senescence, apoptosis, and DNA damage.^209^ Moreover, supraphysiological T can trigger two parallel autophagy-mediated pathways that induce ferroptosis and enable autophagosomal DNA to activate cytosolic nucleic sensor and the innate immune signaling pathway, ultimately increasing the infiltration of immune cells including cytotoxic CD8 cells.^218^

Notably, Cai et al.,^219^ showed that AR mRNA and protein expression are differentially regulated by AR itself in an androgen level-dependent manner. An enhancer within the AR second intron mediates increased AR expression under low androgen levels in CRPC cells, while higher androgen levels repress *AR* gene expression through recruitment of lysine-specific demethylase 1 (LSD1) and H3K4me1,2 demethylation, along with repression of androgen synthesis, DNA synthesis, and cell proliferation. This negative feedback loop within the androgen-AR regulatory axis supports the long-standing observations of a biphasic androgen response. Additionally, a more recent report^212^ demonstrated AR forms monomers under low-dose androgens to drive proliferation via non-genomic mechanisms by activating mTOR signaling and upregulating E2F1 and c-Myc targets. However, high-dose androgen promotes canonical genomic action of the AR to induce the expression of classical AR target genes associated with a differentiated phenotype. This study further complemented the findings of Cai et al. from different biological perspectives of androgen response in prostate cancer cells and provided another mechanistic explanation of ADT resistance due to persistent AR expression and non-canonical AR monomeric functions under castrate levels of androgen. Moreover, another study suggested that canonical AREs with tumor-suppressive functions were depleted in the AR cistromes of prostate cancers, potentially offering an additional explanation for the response to bipolar androgen therapy (BAT).^220^ However, direct profiling of these canonical AREs, along with key intronic and distal enhancers of the *AR* gene, under high-dose androgen conditions has yet to be performed, leaving the question open as to whether BAT reactivates tumor-suppressive regulatory elements in CRPC. These results underscore the pressing need to assess AR structural forms and decode associated AR activities across human PCa, in order to develop novel drugs and therapeutic strategies that can overcome treatment resistance and maximize patient benefit.

The mechanisms of the dose-dependent actions of androgen form the foundation for using testosterone as a therapeutic agent in prostate cancer. While transdermal testosterone, which achieves physiological serum testosterone levels, showed limited efficacy in two independent CRPC cohorts,^221^^,^^222^ the efficacy of BAT—involving rapid cycling from supraphysiological to near-castration testosterone levels over a 4-week cycle —has been evaluated in castration-sensitive prostate cancer patients as well as CRPC patients progressing on ADT or more potent next-generation AR-targeted drugs (e.g., abiraterone or enzalutamide). Across these trials, BAT yielded a favorable response rate of approximately 30%–40% and a median progression-free survival of about 6 months.^223^, ^224^, ^225^, ^226^ Predictive factors for BAT response include high AR expression and/or AR activities (as indicated by canonical AR target gene expression), or mutations in *TP53* or DNA damage repair genes.^227^, ^228^, ^229^ Notably, BAT-mediated AR downregulation may confer resistance to supraphysiological androgen,^209^ yet re-sensitize tumors to AR inhibition in several clinical trials.^224^^,^^226^^,^^227^^,^^230^ Importantly, BAT is primarily studied in minimally symptomatic or asymptomatic participants, since the rises in testosterone could cause an early disease flare and worsening of symptoms.

### 4.4 Molecularly targeted therapies

**Fig. 3:**
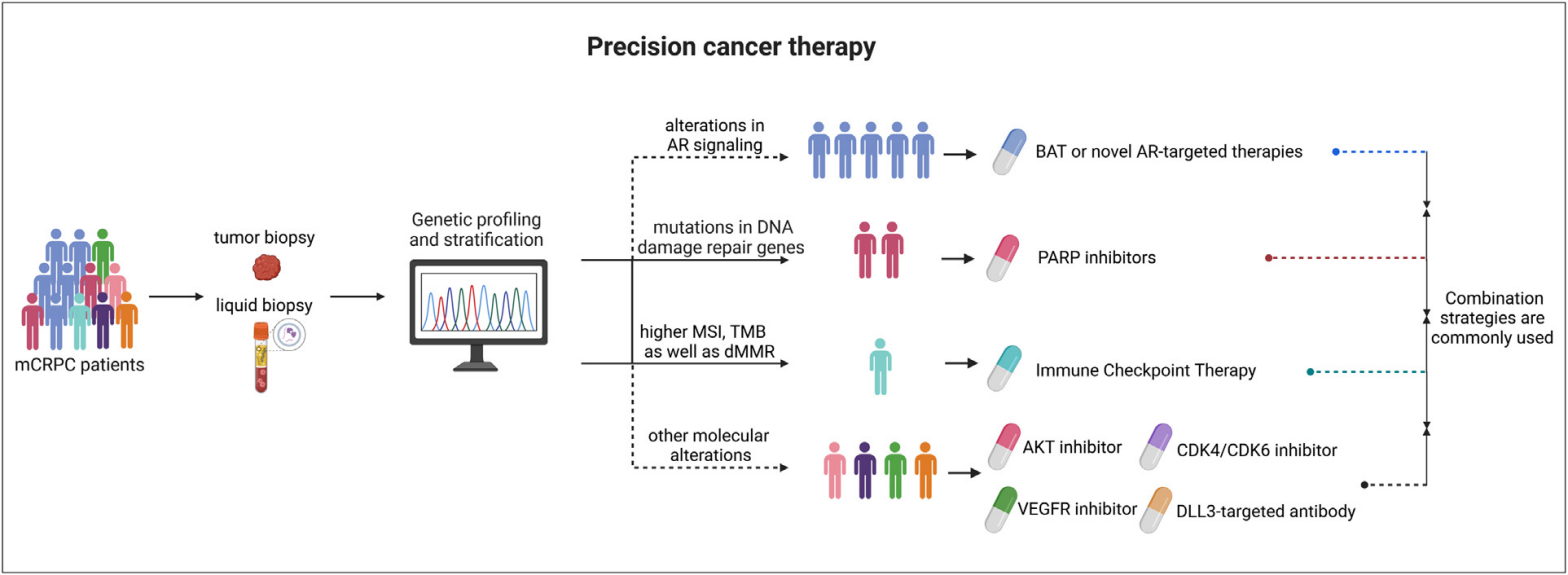
Biomarker-driven approaches for precision cancer therapy. Although ADT or AR antagonists remain the standard-of-care for advanced prostate cancer regardless of specific molecular profiles, genetic stratification of mCRPC patient—using tumor or liquid biopsy testing—can help identify subpopulations harboring actionable biomarkers. These may include DNA damage repair gene alterations suitable for PARP inhibitors, high MSI/TMB or dMMR for immune checkpoint therapy (e.g., pembrolizumab), and other molecular changes (e.g., AKT, CDK4/6, DLL3) that could be addressed by investigational targeted agents. In addition, novel AR/androgen‐targeted therapies (e.g., bipolar androgen therapy, BAT) may benefit patients with AR pathway alterations. Importantly, combination strategies (e.g., PARP inhibitors plus AR inhibitors) are often employed or being evaluated to further improve outcomes. This biomarker‐driven approach aims to personalize treatment and optimize clinical benefit for patients with lethal prostate cancer. ADT, androgen deprivation therapy; AR, androgen receptor; BAT, bipolar androgen therapy; mCRPC, metastatic castration-resistant prostate cancer; MSI, microsatellite instability; PARP, poly(ADP-ribose) polymerase; TMB, tumor mutational burden. Created with Biorender.com.

While AR signaling remains the primary target for treating metastatic prostate cancer—with ADT and potent AR inhibitors (enzalutamide or abiraterone) serving the first-line therapeutic options, advances in genomic sequencing of prostate cancer biopsies have substantially improved our understanding of recurrent genomic alterations across various mCRPC cohorts, thus enabling precision-targeted therapy (Fig. 3).^10^^,^^11^^,^^231^ Apart from AR genomic alterations—which occur in more than 50% of patients with mCRPC—common recurrent gene alterations in CRPC include *TP53* mutations or deletions (> 40% of cases); *PTEN* deletion (in 27%–45% of cases); loss of *RB1* (in ∼10%–20% of cases); alterations in *BRCA2, BRCA1, ATM, CDK12* and other genes involved in homologous recombination (HR) DNA repair (in ∼6%–10% of cases); aberrations in the WNT signaling pathway (in ∼15% of cases); and alterations in genes encoding epigenetic regulators (in ∼10%–20% of cases).^10^^,^^11^^,^^50^^,^^232^ Patients with lethal prostate cancer often exhibit co-occurring alterations in these pathways, and at least one clinical actionable mutation was reported in about 30% of mCRPC patients who also harbor AR alterations.^10^^,^^50^ Notably, an integrative analysis of exome sequencing data from 1,013 prostate cancers identified additional significantly mutated genes that occur less frequently (<5% frequency); their biological and therapeutic implications require further investigation.^233^ Collectively, genomic characterization of the molecular landscape of prostate cancer has greatly improved patient stratification, biomarker-driven precision medicine, and the development of new targeted therapies. Given that many altered signaling pathways are shared with other cancer types, several FDA-approved agents are currently being re-evaluated in clinical trials for prostate cancer management, with some already integrated into clinical practice.

*PTEN* loss leads to hyperactivation of the PI3K-AKT-mTOR pathway,^234^ critical for tumor growth and survival, and is associated with reciprocal feedback activation between AR signaling and PI3K–AKT–mTOR activity.^235^^,^^236^ Specifically, inhibiting one pathway (e.g., PI3K–AKT–mTOR) can drive compensatory upregulation of the other (AR), and vice versa, exacerbating resistance mechanisms.^236^ While monotherapy targeting AKT, PI3K or mTOR yields limited clinical response, this crosstalk indicates that dual inhibition of both the PI3K-AKT-mTOR pathway and AR may be necessary to overcome resistance. Indeed, in the Phase II IPATential150 trial,^237^^,^^174^ significant radiographic progression-free survival (PFS) benefit was observed among patients receiving abiraterone plus the AKT inhibitor ipatasertib for patients who had *PTEN* loss (stratified by immunohistochemistry) (Table 2). Moreover, defects in HR pathways render tumor cells particularly sensitive to poly (ADP-ribose) polymerase (PARP) inhibitors —key modulators of DNA repair—or to DNA damage-inducing agents such as platinum-based chemotherapy, exploiting synthetic lethality.^238^ PARP inhibitors have emerged as a therapeutic strategy for breast and ovarian cancers, especially in patients with germline BRCA1/2 mutations or other defects in DNA damage repair pathways.^239^ Based on the results from several Phase II/III clinical trials that demonstrated promising survival benefits, two PARP inhibitors, olaparib and rucaparib, were FDA-approved in 2020 for the treatment of mCRPC patients harboring genetic alterations in HR DNA repair genes (Table 1). Specifically, olaparib is approved for any mutations in DNA damage repair genes in patients previously treated with abiraterone or enzalutamide (regardless of prior chemotherapy), while rucaparib is approved only for patients with *BRCA* mutations who have already received a taxane and either abiraterone or enzalutamide.^240^^,^^241^ Additionally, ADT treatment can enhance sensitivity to radiation and alter the expression of DNA repair genes, highlighting the functional interplay between androgen signaling and DNA damage repair (DDR) pathways.^242^ In support of this synthetic lethality, multiple combinations of PARP inhibitors and AR pathway inhibitors have been FDA-approved (Table 1), including talazoparib and enzalutamide, olaparib and abiraterone, and niraparib and abiraterone for the treatment of mCRPC with specific defects in the homologous recombination repair (HRR) pathway (Table 1).

Alterations in *RB1* or *TP53* can deregulate cell cycle control, representing the second largest group of mCRPC patients beyond AR alterations.^10^ However, no FDA-approved therapies specifically target *RB1* or *P53* mutations/deletions in mCRPC. CDKs, including CDK4/6, are vital for the G1-S transition and can induce RB1 phosphorylation to reduce its interactions with E2F complex, thereby transactivating E2F transcription factors such as E2F1.^243^ CDK4/6 inhibitors—previously used in hormone receptor-positive/HER2-negative (HER2-) advanced breast cancer^244^ are now evaluated in multiple Phase II/III trials for RB1-expressing mCRPC, either alone or in combination with AR inhibitors.^11^ Current strategies targeting TP53 loss-of-function alterations include restorage of wild-type p53 by preventing MDM2-mediated degradation (e.g., RG7112) or reactivating mutant p53 to adopt a wild-type like conformation.^245^^,^^246^ These p53 modulators have remained largely in preclinical studies or advanced only into Phase I trials.^247^

Notably, co-current loss of two of the three tumor suppressors (i.e., *PTEN, RB1, TP53*)—can shift tumors towards AR independence, promoting lineage plasticity and acquisition of small cell/NEPC histologic and/or molecular features.^239^ Patients with these co-occurring mutations often develop highly aggressive disease, shorter overall survival, and resistance to standard hormonal therapies or AR-targeted agents. They are frequently managed following clinical protocols for small-cell lung cancer (SCLC), such as platinum-based chemotherapy.^239^ The acquisition of NE phenotypes is driven by distinct molecular characteristics, including epigenetic reprogramming, which exposes new therapeutic vulnerabilities. Emerging therapies for CRPC-NE patients have been discovered through combined preclinical and clinical investigations. For example, inhibition of EZH2, a histone methyltransferase that drives global methylation changes in NEPC, restores androgen receptor expression and sensitivity to antiandrogen therapy in preclinical models.^161^ EZH2 inhibitors are currently being investigated in phase I clinical trial (Table 2). Moreover, DLL3 (delta-like ligand 3) is a cell surface protein highly expressed in the majority of NEPC tumors (76.6%) but expressed at low levels in CRPC-Adeno (12.5%), with minimal or absent expression in localized prostate cancer and benign tissues,^248^ suggesting DLL3 is a promising target for NEPC. Although DLL3-targeted antibody-drug conjugates such as rovalpituzumab tesirine (Rova-T) have been investigated in early-phase trials for DLL3-expressing NEPC, further development has been limited due to modest efficacy and toxicity concerns (Table 2). However, the DLL3-targeted bispecific T-cell engager (BiTE) tarlatamab—which has already received FDA approval for SCLC—is currently being investigated for use in neuroendocrine prostate cancer (NCT04702737) (Table 2). Targeting other emerging NEPC derivers, such as pluripotency TF SOX2,^160^, and lineage-associated transcription factors such as BRN2^165^ and N-myc,^11^ holds therapeutic potentials in future clinical trials.

Other therapeutic targets under consideration for mCRPC include WNT signaling and angiogenesis.^231^^,^^249^ Mutations in WNT pathways (e.g., β-​catenin (*CTNNB1*), adenomatous polyposis coli protein (*APC*) and E3 ubiquitin-​protein ligase RNF43 (*RNF43*) affect a significant proportion of mCRPC patients.^50^^,^^231^^,^^232^ However, WNT pathway inhibitors face developmental hurdles due to pathway complexity, crosstalk, and WNT’s essential role in tissues homeostasis, which can lead to systemic toxicity under prolonged WNT blockade.^249^ Anti-angiogenic treatments targeting VEGF/VEGFR have been investigated for CRPC patients. While several Phase III trials evaluating single agent VEGFR inhibition showed limited survival benefit, combining a VEGFA inhibitor with docetaxel improved median PFS and promoted major prostate specific antigen (PSA) response in CRPC.^231^^,^^249^

### 4.5 Immunotherapy

Sipuleucel-T is a dendritic cell (DC) vaccine and currently the only FDA-approved active cellular immunotherapy for asymptomatic or minimally symptomatic mCRPC^250^ (Table 1). It is comprises autologous antigen-presenting cells activated *ex vivo* by a fusion protein (PA202), which links prostatic acid phosphatase to granulocyte-macrophage colony-stimulating factor.^251^ Reinfusion of Sipuleucel-T induces both T- and B-cell-associated sustained immune responses, demonstrates antitumor efficacy, reduces PSA levels, and improves overall survival (OS).^252^ Combinatorial approaches involving Sipuleucel-T plus other approved mCRPC drugs or cytokine are being explored to enhance efficacy. For example, the combination of Sipuleucel-T with cytokine interleukin (IL)-7 has shown significantly increased immune responses and PSA reductions compared with Sipuleucel-T alone.^252^

Immune checkpoint therapies (ICT) targeting programmed cell death-1 (PD-1)/programmed cell death-ligand1 (PD-L1) and cytotoxic T lymphocyte antigen-4 (CTLA-4) have demonstrated clinical benefits and gained FDA approval for various unresectable solid tumors (e.g., metastatic melanoma, non-small cell lung cancer).^253^ However, mCRPC patients typically show *de novo* resistance to these therapies,^254^^,^^255^ and multiple randomized phase III studies have demonstrated the lack of efficacy of ICT when added to standard of care backbone therapies including docetaxel or enzalutamide.^256^, ^257^, ^258^ Prostate cancer is a heterogeneous disease often classified as an “immune cold” tumor —characterized by devoid of T cell infiltration, a lower tumor mutational burden (TMB), a low incidence of mismatch-repair deficiency (dMMR) or microsatellite instability (MSI), an immunosuppressive tumor microenvironment (TME) , and deficient antigen presentation.^259^, ^260^, ^261^ Despite this, the response to CTLA-4-targeting antibodies (e.g., ipilimumab) correlates with high intratumoral CD8^+^ T cell density,^262^ and exceptional responders to pembrolizumab (anti-PD-1) tend to have higher MSI, TMB, and dMMR.^263^ Notably, these genetic alterations occur in about 3%–5% of mCRPC patients,^10^ therefore serving as biomarkers to select those who might benefit from single agent PD-1 inhibition. Preclinical study indicated that an additional predictive biomarker of anti-PD-1 response is biallelic *CDK12* loss, which was linked to a higher neoantigen burden, increased T cell infiltration and clonal expansion.^264^ While mCRPC patients harboring bi-allelic *CDK12* loss showed significant PSA responses to anti-PD-1 monotherapy in initial clinical studies,^264^ a phase II trial of combination ipilimumab and nivolumab (anti-PD-1) or nivolumab monotherapy had negligible activity in patients with mCRPC and deleterious *CDK12* alterations.^265^ Ultimately at this time, ICT is reserved as an option only for patients with mCRPC who harbor tumors with high TMB (> 10 mutations/Mb) or high MSI under the tumor-agnostic approvals of pembrolizumab.^266^^,^^267^

Although androgens have been shown to exert immunosuppressive effects,^268^ the extent to which ADT or AR inhibitors can potentiate ICT responses in mCRPC remain controversial. Emerging preclinical evidence suggests that AR-targeted therapies increase tumor immunogenicity by enhancing immune infiltration (e.g. native T cells and Th-1 cells) into the prostate TME,^269^, ^270^, ^271^, ^272^, ^273^ boosting cytotoxic function, relieving AR-mediated suppression of IFN-γ signaling,^274^ and upregulating MHC-I expression in prostate cancer cells.^275^ The lack of clinical benefit seen in clinical trials could be attributed by complex and dynamic change of TME and transient effect on T cell infiltration following AR inhibition in patients, underscoring further investigation of T cell dynamics and treatment timing to improve responses.^276^ Moreover, combining ICT agents with conventional therapies (e.g., chemotherapy or radiotherapy) or targeted interventions (e.g., olaparib) may boost tumor immunogenicity and MHC-I-dependent antigen presentation, potentially overcoming ICT resistance in CRPC.^277^ Nevertheless, these ICT combination strategies have yet to demonstrate overall survival benefit in mCRPC.^278^ Additional investigation and clinical trials are necessary to evaluate novel combinatorial approaches tailored to individual immune phenotypes or genomic contexts, which will help elucidate the mechanisms underlying both primary and acquired resistance to ICT. Combination of ICT with novel targeted therapies are under investigation in clinical trials (NCT04388852, NCT04471974). Additionally, while traditional ICT agents have not shown benefit in prostate cancer, the anti-B7-H3 antibody enoblituzumab has shown encouraging initial clinical activity in non-castrate patients.^179^ B7-H3 (also known as PD-L3) is an immunomodulatory cell surface protein belonging to the same family as the PD-L1 immune checkpoint protein. It is highly upregulated in prostate cancer, where its expression is associated with poor clinical outcomes and more aggressive disease.^179^^,^^279^ While the putative mechanism of enoblituzumab is antibody-dependent cell-mediated toxicity, there is evidence of a localized immune response—particularly in patients who exhibited T cell clonal expansion following treatment—suggesting that B7-H3 blockade may, in some cases, elicit an immunostimulatory effect.^179^

Additional emerging immune-based therapy for prostate cancer includes novel cancer vaccines, chimeric antigen receptor T cells (CAR Ts), bispecific antibodies, small-molecule tyrosine kinase inhibitors with immune modulatory properties.^252^ Cancer vaccines employ tumor-specific DNA, RNA, or peptide antigens that are highly expressed in malignant vs. normal tissues. Molecules currently used for PCa vaccine development include prostatic acid phosphatase (PAP), PSA, prostate-specific membrane antigen (PSMA), prostate stem cell antigen (PSCA), and six-transmembrane epithelial antigen of the prostate-1 (STEAP1). PROSTVAC is a poxvirus-based PSA-targeting vaccine, which has shown clinical survival benefit in mCRPC patients in a Phase II study.^280^^,^^281^ Though it failed to improve overall survival in a Phase III trial,^282^ it remains a noteworthy example of vaccine-based immunotherapy for mCRPC. Ongoing efforts on several other vaccines platforms have shown immunogenicity and clinical activity in early-phase mCRPC trials.^252^ CAR T therapy uses genetically modified T cells expressing a CAR that recognizes tumor surface antigen independent of MHC (often MHC-I) restriction.^283^ The efficacy of CAR T therapy hinges on antigen selection (e.g., PSCA, PSMA, EpCAM for prostate cancer) and robust T-cell expansion, differentiation, and persistence *in vivo.*^284^ BiTEs simultaneously bind a tumor-specific antigen and CD3 on T cells, bringing T cells into close proximity with tumor cells to induce direct cytotoxic effects.^285^ They can also reactivate T cells by promoting their proliferation independently of MHC or additional co-stimulatory signals.^252^ The anti-STEAP1 BiTE has shown encouraging clinical activity in heavily pre-treated patients with mCRPC, and is currently undergoing evaluation in a phase III trial (NCT06691984).^178^

## 5 Conclusions and future directions

Despite significant therapeutic progress, AR signaling remains the mainstay target for treating prostate cancer. Yet, inevitable resistance to AR-directed therapies—ranging from conventional ADT to next-generation AR inhibitors—continues to challenge long-term clinical outcomes. Accruing clinical evidence suggests that optimizing treatment sequences or combining currently approved agents (e.g., AR inhibitors, chemotherapy, PARP inhibitors, immunotherapies) holds promise to overcome cross-resistance, enhance clinical response, and delay therapeutic resistance.^276^^,^^286^ However, tumor evolutionary dynamics following these various therapeutic approaches can lead to the emergence of new or overlapping drug resistance mechanisms. These may include mutations in drug targets, phenotypic plasticity, feedback-mediated activation of parallel signaling pathways, and the multifaceted consequences of treatment pressure, such as increased intratumoral heterogeneity and clonal selection.^287^, ^288^, ^289^ For example, the complete blockade of AR signaling using emerging AR-targeted drugs (e.g., domain-specific AR inhibitors or AR PROTACs) may increase the percentage of tumors undergoing a phenotypic switch to AR-independent states, for which no targeted therapies are currently available.

Thanks to rapid progress in genomic profiling and mechanistic research, our current understanding of common resistance mechanisms—such as AR mutations, amplifications, splice variants, cistrome reprogramming, or bypass signaling—has greatly expanded, driving the development of more potent AR inhibitors, innovative AR degraders (e.g., PROTACs), non–LBD–directed strategies (e.g., NTD or DBD inhibitors). Moreover, targeting the key enzymes involved in androgen synthesis/conversion pathway can reduce the intra-prostatic androgen bioavailability to inhibit AR oncogenomic activities. Molecular characterization of mCRPC biopsies has, in turn, revealed additional actionable gene defects (e.g., HR DNA repair pathway alterations, *PTEN* loss) that enable precision or targeted therapies (e.g., PARP inhibitors, AKT inhibitors), facilitate effective drug combinations to combat resistance, and may substantially improve outcomes in defined patient subgroups (Fig. 3). While immunotherapy (e.g., Sipuleucel-T, pembrolizumab) can be effective in select cases, further research is needed to determine optimal strategies for immune-based treatments. Acquired resistance emerging in completed or ongoing clinical trials with combination therapies should be further investigated and actively monitored to guide future treatment decisions.

Beyond these current advances, gene editing represents an ever-expanding frontier for directly targeting genetic lesions once deemed “undruggable”.^290^ Notably, CRISPR/Cas13 systems have demonstrated strong preclinical efficacy by silencing oncogenic transcription factor mRNAs in mouse models,^126^^,^^291^ suggesting a new avenue for combating resistance in advanced disease. Looking forward, clinical development of novel agents—including next-generation AR inhibitors, dual or pan-AR degraders, and emerging immunotherapies—will continue at a rapid pace. Biomarker-driven therapeutic strategies, supported by genomic profiling and robust translational research, will become increasingly vital for selecting patients most likely to benefit from specific treatments or combinations (Fig. 3). Ultimately, the goal is to advance precision oncology in advanced prostate cancer, extending survival and improving quality of life for patients who, thus far, have faced limited long-term options.

## Author contributions

**Furong Huang**: Writing. **Kexin Li**: Writing. **Jeffrey W. Shevach**: review & editing. **Qianben Wang**: review & editing.
